# The Effect of Prolonged Culture of Chromosomally Abnormal Human Embryos on The Rate of Diploid Cells 

**DOI:** 10.22074/ijfs.2015.4617

**Published:** 2015-12-23

**Authors:** Masood Bazrgar, Hamid Gourabi, Poopak Eftekhari-Yazdi, Hamed Vazirinasab, Mostafa Fakhri, Fatemeh Hassani, Mohamad Chehrazi, Mojtaba Rezazadeh Valojerdi

**Affiliations:** 1 Department of Genetics, Reproductive Biomedicine Research Center, Royan Institute for Reproductive Biomedicine, ACECR, Tehran, Iran; 2 Department of Developmental Biology, University of Science and Culture, Tehran, Iran; 3 Department of Embryology, Reproductive Biomedicine Research Center, Royan Institute for Reproductive Biomedicine, ACECR, Tehran, Iran; 4 Department of Epidemiology and Reproductive Health, Reproductive Epidemiology Research Center, Royan Institute for Reproductive Biomedicine, ACECR, Tehran, Iran

**Keywords:** Aneuploidy, Blastocyst, Self-Correction, Mosaicism

## Abstract

**Background:**

A decrease in aneuploidy rate following a prolonged co-culture of human blastocysts has been reported. As co-culture is not routinely used in assisted reproductive technology, the present study aimed to evaluate the effect of the prolonged single culture on the rate of diploid cells in human embryos with aneuploidies.

**Materials and Methods:**

In this cohort study, we used fluorescence in situ hybridi- zation (FISH) to reanalyze surplus blastocysts undergoing preimplantation genetic diagnosis (PGD) on day 3 postfertilization. They were randomly studied on days 6 or 7 following fertilization.

**Results:**

Of the 30 analyzed blastocysts, mosaicism was observed in 26(86.6%), while 2(6.7%) were diploid, and 2(6.7%) were triploid. Of those with mosaicism, 23(88.5%) were determined to be diploid-aneuploid and 3(11.5%) were aneuploid mosaic. The total frequency of embryos with more than 50% diploid cells was 33.3% that was lower on day 7 in comparison with the related value on day 6 (P<0.05); however, there were no differences when the embryos were classified according to maternal age, blastocyst developmental stage, total cell number on day 3, and embryo quality.

**Conclusion:**

Although mosaicism is frequently observed in blastocysts, the prolonged single culture of blastocysts does not seem to increase the rate of normal cells.

## Introduction

Chromosome abnormality is a frequent phenomenon in the human preimplantation stage, according to reports of analyzed embryos (1-3). The aneuploidy rate of the cleavage stage is various depending on techniques and number of analyzed chromosomes (2). On average, 60% abnormality has been reported in a review of fluorescence in situ hybridization (FISH) studies (4), while more than 90% of embryos have been abnormal after examining all blastomeres using microarrays analysis (1, 5). Routinely, one blastomere is biopsied on the third day post-fertilization; however, sometimes it is necessary to biopsy two blastomeres. In such cases, disagreement between the results of the two analyzed blastomeres is considered as a case of mosaicism which is identified as the presence of two or more genetically different cell lines in an embryo. Mosaicism is a highly frequent phenomenon during the cleavage stage because the majority of cell division errors in early embryos occur during this stage. Due to inactivation of the genome during human embryo fertilization, genome stability until the third cell division is mainly controlled by cytoplasmic transcriptomes of the oocyte. Degradation of mRNA in oocyte decreases fidelity of the cell division because genome activation in the human embryo mainly occurs after the third cell division (6). Therefore, preimplantation abnormalities are mainly post zygotic events that arise by error-prone cell division during inactive status of embryonic genome (7).

While clinical studies of blastocyst aneuploidy are limited, these reports have shown approximately 60% aneuploidy in blastocyst stage, of which 33% are mosaic. Of these, some are diploid- aneuploid and some aneuploid mosaic (8). Numerous reports have shown that reanalyzed embryos at the blastocyst stage, with aneuploidy on the third day of development, mostly achieve full diploidy by less than 18% (2, 3, 9-12). However, these reports have predominantly focused on day 5 blastocysts. A comparison of the aneuploidy rate in days 4, 5 and 8 of embryos co-cultured with an endometrial layer has shown an increased rate of normal cells in the analyzed embryos after increasing culture time (13). Munne et al. (14) have co-cultured aneuploid embryos with fibroblasts and analyzed these embryos on days 6 and 12. According to their results, there was an increase in the rate of normal cells to 48% by day 12. Numerous studies report derivation of normal human embryonic stem cells (hESCs) from embryos detected as aneuploid in the cleavage stage (15-18) and blastocyst stage (19). The establishment of hESC lines is routinely performed by being co-cultured with a feeder layer. While mosaicism is highly frequent in early embryos, an increase in the rate of normal cells seems to be a progressive phenomenon by additional embryonic development because of their growth advantage rather than aneuploid cells (20).

This preliminary study aimed to evaluate the effect of prolonged culture on diploidy rate. We increased the culture time to days 6 and 7 as the last days before closing of the implantation window. Numerous reports from day 5 of development in spare embryos have shown low percentage of full diploidy in analyzed blasocysts (2, 3, 9-12), while after being co-cultured for 8-12 days, there is an increase in percentage of normal cells (13, 14). As single culture is more routinely used than coculture in assisted reproductive technology (ART), we preferred to use single culture for the embryos in order to evaluate diploidy rate.

## Materials and Methods

This cohort study was approved by the Ethics Committee of Royan Institute, Tehran, Iran, and performed on spare embryos from preimplantation genetic diagnosis (PGD) candidates, who signed an informed consent. We used simple random sampling method to include the study group.

### Sample preparation

In this study, inclusion criteria were as follows: stimulation by the long protocol described previously (21) and fertilization by intra-cytoplasmic sperm injection (ICSI). The embryos were cultured in sequential media (Vitrolife, Sweden) under mineral oil (Origio, Denmark). Following routine ART treatments, two pronucleate (2PN) zygotes were transferred to fresh microdrops of G-1^TM^ V5 medium (Vitrolife, Sweden) supplemented with 10% human serum albumin (Vitrolife, Sweden). The embryo biopsy for PGD was performed 72 hours after fertilization. In order to perform an embryo biopsy on day 3, we incubated the embryos for 1-2 minutes in Ca2+/Mg2+-free G-PGD^TM^ biopsy medium (Vitrolife, Sweden). After the biopsy of one blastomere for PGD, the embryos were transferred to G-2^TM^ V5 medium (Vitrolife, Sweden), while those selected for freezing, either aneuploid or unsuitable, underwent a prolonged culture. We cultured 100 spare embryos of PGD candidates for 6-7 days postfertilization.

### Spreading of the blastocysts

Each embryo reaching the blastocyst stage was randomly spread on day 6 or 7 of development. We performed the spreading process according to previously described procedure (22) with some modifications. Embryos were briefly washed in two drops of phosphate buffer saline (PBS, Gibco, USA), then transferred to 1 mM/L HCl (Merck, USA)-1% Tween 20 (Sigma-Aldrich, USA). After 2-4 minutes, the embryos were transferred to a glass slide with less than 1 μl HCl-Tween 20. When necessary, we added additional HCl-Tween 20 to complete spreading. The slides were allowed to air dry for 45 minutes, after which they were washed in PBS for 5 minutes and dehydrated in a graded ethanol series of 70, 85 and 100%.

### Fluorescence in situ hybridization

The slides were pre-treated with pepsin (Sigma- Aldrich, USA, 400 μg/ml) in 0.1N HCl at 37˚C, then fixed in 10% formalin (Merck, USA) at 4˚C and washed in PBS at room temperature (each step for 5 minutes), after which slides were treated by 2X standard saline citrate (SSC) for 10 minutes at 37˚C. Slides were again fixed in formalin and rewashed in PBS, dehydrated in another graded series of ethanol (70, 85 and 100%), and allowed to air dry. Chromosome aneuploidies were studied in two rounds by FISH using the locus-specific identifier (LSI) 13, chromosome enumeration probe (CEP) 18, LSI 21, LSI 22, CEP 15, CEP X and CEP Y probes (Vysis, USA). Following heat denaturation of the nuclear and probes’ DNAs at 75˚C for 5 minutes, the hybridization step was performed by incubation of the slides at 37˚C, overnight. The next day, slides were washed in 0.4X SSC/0.3% NP-40 (Vysis, USA) at 72˚C for 2 minutes that was followed by immediate washing in 2X SSC/0.1% NP-40 for 5 minutes at room temperature. After the nuclei were stained with 4´, 6-diamidino-2-phenylindole (DAPI), we analyzed only cells with interpretable signals from each blastocyst. For analysis, we used a Nikon fluorescent microscope (Nikon, Japan) equipped with appropriate filters that could detect FISH signals. In the first round of FISH, the position of the nuclei on the slide was recorded by a schematic drawing to enable recording the results of the second round. FISH signals were scored as previously described (23).

### Embryo classification

Embryos were classified according to the following characteristics: day of reanalysis (days 6 or 7 post-fertilization); stage of blastocyst reanalysis (hatched or earlier stages of the blastocyst development); numbers of total cells on day 3; maternal age (<37 or ≥37 years); indications for PGD; and quality of embryos on day 3 according to their fragmentation pattern and morphological characteristics, including blastomeres compaction, equal size, absence of vacuoles, presence of multinuclei and granularity of cytoplasm as previously described (24). Regarding very low incidence of fully diploid blastocysts, comparisons was performed between categories of more and less than 50% normal cells.

### Statistical analysis

Data analysis was performed using the SPSS (version 16.0, SPSS Inc., USA) statistical software. The logistic regression models with sequential and variable selection were constructed using Hosmer–Lemeshow test (25). P<0.05 was considered significant.

## Results

In this study, among 100 embryos from 19 patients, 30 reached the blastocyst stage. Table 1 presents some embryological data of these patients. The fertility rate was 70.4% and the overall maternal age was 33.9 years (range 25-40 years).

Totally, 293 nuclei from 30 blastocysts were included in data analysis; the mean number of nuclei per embryo was approximately 10 (range 3-17). It is noted that we included data regarding the cells with interpretable signals in both FISH rounds.

**Fig.1:**
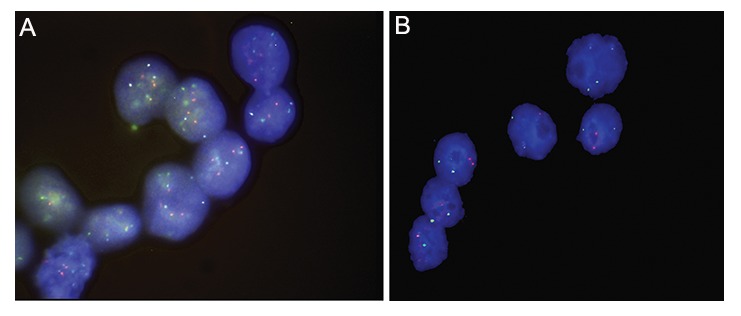
Mosaicism in nuclei of blastocysts after the first round of fluorescence in situ hybridization (FISH) showing chromosomes 13 (green), 18 (aqua), 21 (red), DNA stained with 4´, 6-diamidino-2-phenylindole (DAPI). A. Tetraploid-diploid mosaicism and B. Aneuploid mosaicism.

In primary analysis of these 30 embryos on day 3, frequencies of aneuploid and diploid embryos were 21(70%) and 7(23.3%), respectively. Two (6.7%) out of 30 embryos had no results on day 3, while they were diploid-aneuploid mosaic regarding blasocyst analysis. Of these, one was mosaic diploid-tetraploid (Fig .1A) that tetraploidy was observed in 5 out of 17 analyzed cells (29.4%, Table 2). By reanalysis of 21 aneuploid embryos, 1(4.7%) with triploidy on day 3 showed triploidy again, whereas 1(4.7%), 3(14.3%) and 16(76.2%) were diploid, aneuploid mosaic and diploid-aneuploid mosaic, respectively. Of 7 diploid embryos on day 3, only 1(14.3%) showed diploidy upon reanalysis of the blastocyst stage, while 1(14.3%) and 5(71.4%) were triploid and diploid-aneuploid mosaic, respectively.

The most frequent abnormality in the analyzed blastocysts was mosaicism observed in 26(86.6%) embryos, of which 23(88.5%) were diploid-aneuploid mosaic. The total frequency of diploid-aneuploid mosaicism among the analyzed embryos was 76.6%. Mosaic aneuploidy was observed at a frequency of 10%, there is no diploid cell in the embryos with mosaic aneuploidy. Concordance of FISH results of all analyzed cells from each blastocyst with primary analysis on day 3 were remarkable for 4(13.3%) embryos, where 2(6.7%) were diploid and 2(6.7%) were triploid.

The total frequency of blastocysts with more than 50% diploid cells was 33.3%, 10 embryos. The distribution of embryos into categories of more and less than 50% normal cells did not show significant difference when they were classified according to total cell number on day 3, maternal age, developmental stage of the blastocyst, indications for PGD and embryo quality on day 3. The frequency of blastocysts with over 50% normal cells on day 6 was significantly more than those analyzed on day 7, 7 out of 13(53.8%) versus 3 out of 17(17.6%) (Table 3).

Although we did not find a significant difference in distribution of relatively normal embryos according to their total cell numbers on day 3, embryos lagging behind in cell divisions showed higher normalization. The frequencies of embryos with more than 50% normal cells were 62.5% (5 out of 8) versus 23.8% (5 out of 21) for embryos with 5-6 and 7-8 cells on day 3, respectively (P=0.08).

The rate of normal cells in the studied blastocysts was not different between infertile and presumed fertile patients concerning indications for PGD (Table 3).

**Table 1:** Embryological data of the patients with analyzed blastocysts


Patient number	Oocytes	MII oocytes	2PN embryos	Biopsied embryos	Transferred embryos

1	12	10	5	4	1
2	14	12	9	9	4
3	*	*	*	7	3
4	12	11	5	4	1
5	7	7	6	5	2
6	*	*	*	8	3
7	10	10	3	4	2
8	18	18	15	8	4
9	10	9	6	5	1
10	12	12	9	11	3
11	11	10	8	7	1
12	6	5	5	5	1
13	13	13	12	8	1
14	10	9	5	5	2
15	7	6	5	5	2
16	9	7	5	5	2
17	7	7	6	6	2
18	10	8	5	4	1
19	14	13	9	6	3
Mean	10.7	9.8	6.9	6.1	2


*; Missed due to using thawed embryos, MII; Metaphase II and 2PN; Two pronucleate.

**Table 2:** Fluorescence in situ hybridization (FISH) results of embryos in the cleavage and blastocyst stages


Embronumber	Patient number	Day 3 results of single blastomere analysis	Analyzed cells in blastocyst (n)	Aneuploidies in blasyocyst cells	Diploid cells in blastocysts (%)	Blastocyst classification

1	1	Triploid	3	3N[3]	0	Triploid
2	1	+18	16	+18[2] -18[6](6) --18[7] --13[3] -13[4]+13[3] +X[1] -Y[2] +Y[1] -15[1] ++15[1] --21[3] -21[5] -22[2]	6	Mosaic diploidaneuploid
3	2	-18	14	-13[3] +13[2] ++13[2]+++13[1] -15[9] -18[5]	14	Mosaic diploid- aneuploid
		-21		+18[5] ++18[3] --21[1]-21[1] +21[2] ++21[1]		
		-22		--22[8]-22[1] ++X[11]		
4	2	-22 --18	9	Diploid	100	Diploid
5	3	Diploid	7	--13[2] -13[1]	14	Mosaic diploid- aneuploid
6	4	-21 -22	6	-13[4] ++13[1] -18[1]+18[1]-21[1] -21[1]	0	Mosaic aneuploid
7	5	--18 -21	6	-18[3] ++18[1] ++21[4]-22[1] ++22[1]	33	Mosaic diploid- aneuploid
		-22				
8	6	-18 XO	7	--13[1] 13[1] +13[1]-15[1] +15[1]-18[2]-21[1] +21[1] ++21[1]XY[1] XX[1]	29	Mosaic diploid- aneuploid
9	6	Diploid	7	++13[1] +15[1] ++15[1]-21[1] ++21[1]	71	Mosaic diploid- aneuploid
10	6	+13 –15	5	+21[1]	80	Mosaic diploid- aneuploid
11	7	+15	3	-15[1] ++X[1]	67	Mosaic diploid- aneuploid
12	7	+21	6	-15[1] ++15[1] +++15[1]++++15[1]+X[1] ++X[2] +++X[1]	33	Mosaic diploid- aneuploid
13	8	-15	17	-18[10] +Y[1]	35	Mosaic diploid- aneuploid
14	9	Diploid	17	--13[10] -13[5] -15[1]--21[5] -21[1]	12	Mosaic diploid- aneuploid
15	9	+13 +18++21 XO	7	--13[1] -13[1] ++15[1]-21[1] -21[1] +21[1]XY[1] XXYY[1]	43	Mosaic diploid- aneuploid
16	10	-18 XO	6	-13[2] ++15[1] XY[2]	25	Mosaic diploid- aneuploid
17	10	-15 -18	9	-13[7] ++21[3]++18[3]	0	Mosaic aneuploid
18	10	No result	7	-13[1] -15[1] -X[1]+X[1] -Y[1] +Y[1]	25	Mosaic diploid- aneuploid
19	10	No result	17	++13[5] ++18[5]++15[5]++21[5]++X[5]++Y[5]	71	Mosaic diploid- aneuploid (diploid- tetraploid)
20	10	Diploid	12	--13[1] -13[6] –15[1]-18[2] -18[4] -21[2]-21[6]	8	Mosaic diploid- aneuploid
21	10	Diploid	4	+13[4] +18[4] +21[4]	0	Triploid
22	11	Diploid	13	-15[4] -21[1]	67	Mosaic diploid- aneuploid
23	12	+18 ++X	13	-13[1] +15[2]	78	Mosaic diploid-
		+Y		-18[1]		aneuploid
24	13	Diploid	4	Diploid	100	Diploid
25	14	-13	10	-13[3] -18[4] -21[1]-21[1]	30	Mosaic diploid- aneuploid
26	15	+18	11	-18[1] -21[4] -22[1]-X[5] -Y[1]	33	Mosaic diploid- aneuploid
27	16	+18	15	--15[2] -15[1] +15[2]++15[1] XXYY[10]	53	Mosaic diploid- aneuploid
28	17	-15	17	-13[1] ++13[1] -21[3]++21[1]-18[1] +18[2] ++18[1]	92	Mosaic diploid- aneuploid
29	18	+13 +18	16	--13[2] -13[4]--15[1]+15[1]--21[2] -21[4] -X[1]+Y[5]	31	Mosaic diploid- aneuploid
30	19	-18 --21 -22	9	--18[1] -18[2] +18[1]-21[3] -21[4] +21[1]-22[1] -22[3]	0	Mosaic aneuploid


Digits in brackets indicate the numbers of cells that had aneuploidy mentioned before the bracket. -, +; Decrease or increase in number of chromosomes.

**Table 3:** Results of multivariable logistic regression model of relationship between 6 explanatory variables and the relatively normalization (more than 50% normal cells) in blastocysts


Classification criteria		Relatively normal blastocysts	Total number of blastocysts	Odds ratio	95% confidence interval	P value

Day of reanalysis	7	3	17	0.15	0.02, 0.93	0.04
	6	7	13			
Day 3 total cell number	7-8	5	21	0.19	0.03, 1.28	0.08
	Other	5	9			
Stage of reanalysis	Hatched	6	22			
	Other	4	8	1.66	0.15, 17.76	0.67
Maternal age (Y)	≥37	4	9			
	<37	6	21	0.79	0.03, 18.74	0.88
Day 3 quality	Excellent to good	8	21	7.60	0.42, 137.69	0.17
	Fair to poor	2	9			
Indication for PGD	Recurrent miscarriage	2	8	0.40	0.01, 10.07	0.58
	Recurrent implantation failure	3	9	0.89	0.08, 9.18	0.92
	Family Balancing	5	13			


PGD; Preimplantation genetic diagnosis.

## Discussion

In the current study by reanalysis of spare embryos from PGD candidates, we found a variety of abnormalities in blastocysts that could not be diagnosed on day 3 of development analysis of single blastomeres from the same embryo. Regarding high frequency of mosaicism in reanalyzed blastocysts, it seems that the majority of them have been mosaic in cleavage stage, while they could not be diagnosed due to limitation in number of available cell for biopsy at the cleavage stage.

We used FISH as a widely applied technique, like similar research studies (2, 3, 9-12), and preimplantation genetic screening (PGS); however, the results were in agreement with array-based studies and both approaches indicated highly frequency of mosaicism in early embryos. Of note, the majority of blastocysts in the current study were spare aneuploid embryos according to PGSdefined single blastomere from third day, while above-mentioned array-based studies (1, 5) found extensive mosaicism in good quality embryos. It has indicated that mosaicism is common in early embryos even those with good quality; however, blastocysts with aneuploidies on day 3 might contain more abnormal cells and/or more variation of abnormalities among cells. Array-based analysis has the power for analysis of all chromosomes. It is clear that analysis of all chromosomes could result in finding more abnormalities than studying of some chromosomes by FISH. However, in the current study, analysis of 7 chromosomes by FISH showed high frequency of mosaicism and no advantage for prolonged culture of the blastocysts.

We found a higher normal cell rate in embryos analyzed on day 6 compared to the related value on day 7. An increase in the rate of aneuploid cells on day 7 compared to the related value on day 6 in single culture may be caused by longer time exposure to *in vitro* conditions. Our first assumption for this study was to see more normalization during prolonged single culture, while by preliminary analysis of 30 blastocysts, we found a high rate of abnormalities. There are few reports about culture of embryos longer than 6 days that co-culture have been used until day 13 post-fertilization, whereas we used single culture and limited the culture prolongation to day 7 as the last day for embryo transfer before closing the implantation window (26). A recent study has searched the normal growth rate of human embryos between days 3 and 13 in either continues culture or co-culture with mouse embryonic fibroblasts. Their results have showed a higher rate of normalization in day 7 aneuploid embryos as compared with related values of days 5-6 and later up to 13. This study has concluded that normalization occurs mainly until days 7 and 8, whereas longer cultures might lead to a decrease in normalization rate (12), which is in agreement with our findings. However, we could not exactly compare our results with that study because their results are a combination of single cultured and cocultured as well as arrested embryos.

In a similar study by Santos et al. (13) who compared days 4, 5 and 8 embryos, there was an increase in the rate of normal cells by prolongation (6% on day 4, 37% on day 5 and 58% on day 8). They studied embryos co-cultured on endometrial stromal monolayer cells. A recent study on all blastomeres of 13 good quality embryos on day 4 using array-comparative genomic hybridization showed 16-100% abnormal blastomeres in studied embryos. The authors have supposed that fully normalization might occur in later stages of development (5). This phenomenon could happen through several mechanisms for overcoming on aneuploidies, leading to an increase in the rate of diploid cells in mosaic embryos (6).

Munne et al. (14) have reported an increased rate of normal cells in embryos cultured with a fibroblast feeder layer in order to establish hESC lines from aneuploid embryos. A hypothesized reasons for derivation of normal cell lines from aneuploid embryos are the misdiagnoses by the FISH technique due to its limitation and the diagnosis of aneuploidy based on only single blastomere analysis (15). If all normal hESC lines established from day 3 aneuploid embryos have been misdiagnosed, this hypothesis could not answer the establishment of hESC lines from aneuploid blastocysts (19). Furthermore, diagnosis of aneuploidy in blastocyst stage is based on analysis of several cells. A decrease in the rate of abnormal cells might be related to the effects of co-culture of embryos with differentiated cells due to a mimic of implantation. Differentiation is known to be a barrier for the division of aneuploid cells (27). Communications between differentiated cells that have been used for co-culture and embryonic cells might induce some cellular and molecular mechanisms, leading to decrease in the rate of aneuploidies in the embryo. While aneuploidies are considered as an incident in early embryonic development, some aneuploid embryos would be arrested in their development to the later stages. Although aneuploidies incidence would be decreased by reaching to blastocyst stage, mosaic embryos mostly reach to blastocyst stage. Implantation is a critical stage that blastocysts should pass it after hatching. There is not any direct evidence on the effect of aneuploidies on implantation potential, but one of the main reasons for including into PGS is recurrent implantation failure. As the current study was designed for clinical benefits, we studied the embryos without being co-cultured.

The relationship between abnormal morphology on the third day of embryo development and chromosomal abnormalities has been well documented. The abnormal rate of development also correlates with aneuploidies (28). However, we found no significant association between the rate of aneuploid cells in blastocysts to their quality and total cell number on day. Maternal age as another factor for aneuploidy in the cleavage stage (8) showed no correlation with the rate of aneuploid cells in blastocysts.

Although chromosomal abnormalities are known as a cause of infertility, in our study, blastocysts from candidates for family balancing did not show higher rates of normal cells in comparison with blastocysts of infertile patients. This finding is in agreement with a recent study in presumed fertile and infertile patients (29).

A limitation for day 3 PGD is the "no result" cases, meaning that in this study, 6.7% of analyzed embryos were unable to be diagnosed on day 3 PGD, while by availability of a number of cells at the blastocyst stage, we observed decreasing the "no result" rate. Recently, array-based PGD has been more considered due to their ability to screen abnormalities in all chromosomes (5, 30, 31).

Mosaicism, in particular diploid-aneuploid, is a common phenomenon in the blastocyst stage (32). We observed a high frequency of diploid-aneuploid mosaicisms in the current study. Growth advantage of diploid cells in mosaic diploid-aneuploid embryos have been speculated as one reason for overcoming on aneuploidy, because of increased death and decreased division rate in the aneuploid cells (13, 20).

There are three destinations for mosaic embryos following differentiation: abortion, birth defects or healthy newborn. We recently showed that the dominant response to DNA damage in poor-quality pre-implantation human embryos with complex aneuploidy is DNA repair rather than cell division or apoptosis (33). Self-correction could rarely occur in mosaic diploid-aneuploid embryos by advantage of diploid cells for survival and division (5).

A disadvantage for current array-based PGD in the blastocyst stage is the increased time needed to conduct an analysis using array technologies compared with FISH. With regards to the limited time for embryo transfer before closing the implantation window (26), an approach could be embryo vitrification and their transfer in the subsequent menstrual cycles. It should be mentioned that IVF outcomes may be improved by transferring frozen embryos compared with fresh embryos (34). Another concern could be survival of biopsied blastocysts after vitrification; the results of this approach indicated that the implantation rate is comparable with thawed blastocysts, without biopsy (35). Another plan would be performing a biopsy in frozenthawed embryos prior to embryo transfer (36).

We have observed tetraploid-diploid mosaicism in 1(3.3%) embryo (embryo no.19). This event has also been reported during the blastocyst stage, as a result of synchronization of the cell divisions during this stage. This could be considered as a normal status for an embryo. Transfer of an embryo with a tetraploid karyotype on trophectoderm biopsy has resulted in a normal pregnancy (37).

## Conclusion

Mosaicism is frequent in human blastocysts. Cleavage stage PGS does not show extensive aneuploidies in the embryo because of the limited number of biopsied cells. The blastocyst stage could be a good stage for aneuploidy screening by performing an analysis of several cells. Although the longer time co-culture of human embryos has been reported to decrease aneuploidy rate, we did not find any advantage in single culture of blastocysts until day 7. Of note, omission of a co-culture in the current study was to evaluate the clinical benefits of prolonged single culture. It seems for PGS, biopsy of the embryos upon reaching to blastocyst stage and their analysis for selection of normal embryos is better than later biopsies.
